# Differential *In Vitro* Kinetics of Drug Resistance Mutation Acquisition in HIV-1 RT of Subtypes B and C

**DOI:** 10.1371/journal.pone.0046622

**Published:** 2012-10-03

**Authors:** Rodrigo D. Cunha, Celina M. Abreu, Luis M. F. Gonzalez, Monique Nijhuis, Dorien de Jong, Renato S. Aguiar, Adriana O. Afonso, Rodrigo M. Brindeiro, Amilcar Tanuri

**Affiliations:** 1 Laboratório de Virologia Molecular, Universidade Federal do Rio de Janeiro, Rio de Janeiro, Rio de Janeiro, Brazil; 2 Department of Virology, Medical Microbiology, University Medical Center Utrecht, Utrecht, The Netherlands; McGill University AIDS Centre, Canada

## Abstract

**Background:**

HIV-1 subtype B is the most prevalent in developed countries and, consequently, it has been extensively studied. On the other hand, subtype C is the most prevalent worldwide and therefore is a reasonable target for future studies. Here we evaluate the acquisition of resistance and the viability of HIV-1 subtype B and C RT clones from different isolates that were subjected to *in vitro* selection pressure with zidovudine (ZDV) and lamivudine (3TC).

**Methods/Principal Findings:**

MT4 cells were infected with chimeric virus pseudotyped with RT from subtype B and C clones, which were previously subjected to serial passage with increasing concentrations of ZDV and 3TC. The samples collected after each passage were analyzed for the presence of resistance mutations and VL. No differences were found between subtypes B and C in viral load and resistance mutations when these viruses were selected with 3TC. However, the route of mutations and the time to rebound of subtype B and C virus were different when subjected to ZDV treatment. In order to confirm the role of the mutations detected, other clones were generated and subjected to *in vitro* selection. RT subtype B virus isolates tended to acquire different ZDV resistance mutations (Q151M and D67N or T215Y, D67D/N and F214L) compared to subtype C (D67N, K70R, T215I or T215F).

**Conclusions/Significance:**

This study suggests that different subtypes have a tendency to react differently to antiretroviral drug selection *in vitro.* Consequently, the acquisition of resistance in patients undergoing antiretroviral therapy can be dependent on the subtypes composing the viral population.

## Introduction

Human immunodeficiency virus type 1 (HIV-1) can be segregated into several groups, subtypes, sub-subtypes and circulating recombinant forms (CRF) as a consequence of its genetic diversity [1]. While subtype B predominates in the developed world, other non-B subtypes or CRF are responsible for most of the infections occurring in developing countries [2]. Of note, subtype C is responsible for over 60% of global HIV-1 infections, as this variant can be found in countries with the highest known prevalence in sub-Saharan Africa and in highly populated countries such as India and China [2].

HIV-1 resistance is the major virologic factor contributing to therapeutic failure [3]. Many resistance mutations have already been characterized including three multi-drug resistance profiles (insertions at codon 69, Q151M-mediated multinucleoside resistance and thymidine analogue mutations (TAM)). Q151M-associated mutations confer resistance to all nucleoside reverse transcriptase inhibitors (NRTIs) except for tenofovir. TAMs are selected by zidovudine (ZDV) and stavudine (d4T), and impact resistance to all NRTIs [4]. Two distinct TAM resistance pathways can be observed: TAM-1 (M41L, T210W and T215Y) and TAM-2 (D67N, K70R, T215F and K219Q) [5].

HIV-1 subtypes may have different biological characteristics, and may respond differently to diagnostic, immunologic and therapeutic interventions [6]. In regard to HIV antiretroviral (ARV) treatment, several studies have shown that HIV-1 subtype-specific differences influence the *in vitro* susceptibility as well as the resistance mutations selected upon treatment with specific drugs [7]–[9]. Furthermore, HIV-1 subtypes may also differ in the rates of mutation selection and fixation during ARV exposure [10].

In this work we studied the *in vitro* behavior of viruses carrying subtype B or C RT under the selective pressure of two NRTIs, ZDV and 3TC. We could observe a different mutational pattern upon ZDV exposure for subtype B RT variants (TAM-1 pathway; Q151M complex mutations) as compared to subtype C RT variants (TAM-2 pathway). On the other hand, 3TC displays a similar mutational behavior among these two subtypes.

## Materials and Methods

### *In vivo* Recombinant Virus Generation and *in vitro* Selection (RT_B_ and RT_C_ Virus)

A recombinant virus assay technology was used to generate two identical viruses differing solely in the RT palm-finger region (RT codons 35–225). Donor subtype B sequence was obtained from the pNL43 infectious clone. Subtype C sequence belongs to drug-naïve isolate from Brazil [11]. Several differences in the RT gene sequence could be observed between these isolates (V35T, E36A, T39D, K43R, S48T, V90F, Q102R, D121Y, K122E, D123N, S134I, I135T, C162S, E169K, K173N, Q174K, D177E, T200A, Q207E, R211K, and F214L); none of them were previously related to NRTI resistance (Table 1). Samples from RT gene fragment amplification were co-transfected into MT4 cells (CD4+ T lymphocyte lineage, NIH-USA) with the ΔDNApolimeraseRT HXB2 *Bst*EII-linearized plasmid carrying RT deleted (ΔRT) HIV-1 HXB2 genomic DNA, generating a chimeric virus by homologue recombination [12]. Before starting the drug selection, sequencing was performed to confirm the absence of drug resistance mutation (DRM) as well as the integrity of the RT gene. At day 7 post-transfection, supernatants were collected, frozen and used for tissue culture infectious dose 50% (TCID_50_) determination. Recombinant viruses carrying RT from either subtype were evaluated *in vitro* for the kinetics of the acquisition of mutations leading to 3TC and ZDV resistance. HIV-1 molecular subtype B and C chimeric clones (RT_B_ and RT_C_) were used to infect MT4 cells during the selection process with a multiplicity of infection (MOI) of 0.001. Cells were subsequently resuspended in RPMI medium supplemented with 10% FBS and 0.001 µM ZDV or 0.020 µM 3TC graciously donated by NIH. Drug concentration was increased two-fold after each passage until it completely inhibited virus replication. The viral load (VL) was estimated by Taq Man® real time PCR using a quantitative RT-PCR reaction as previously described [13]. The viral RNA was extracted from the culture and cDNA synthesis was performed. The PCR target was located in the U5 region of HIV-1 5′ LTR region using HIV-1 specific oligonucleotides and probe [13]. Calibration curves were generated using a HIV-1 subtype B supernatant with a known VL (10^6^, 10^5^, 10^4^, 10^3^, and 10^2^ viral particles/mL). At least three dilutions of every sample were assayed, and data sets in which the linear correlation coefficient of the standard curve was less than 0.98 were discarded.

**Table 1:** Summarized data of chimeric virus utilized in *in vitro* selections.

RT Origin	RT Subtype	RT fragment codons	Vectors used	Virus production methodology	Virus name	Replicates	Amino acids differences between RT subtype B and C
NL4-3	B	35–225	ΔDNA polimerase RT HXB2 (11)	recombination	RT_B_	1	Reference
patient	C	35–225	ΔDNA polimerase RT HXB2 (11)	recombination	RT_C_	1	V35T, E36A, T39D, K43R, S48T, V90F, Q102R, D121Y, K122E, D123N, S134I, I135T, C162S, E169K, K173N, Q174K, D177E, T200A, Q207E, R211K, F214L
NL4-3	B	25–554	ΔRT HXB2 (13)	cloning	RT_B′_	6	Reference
C23 patient	C	25–315	ΔNRT HXB2 (14)	cloning	RT_C′_	6	V35T, E36A, T39D, K43R, S48T, Q102K, D121Y, K122E, C162S, K173N, Q174K, D177E, T200A, Q207E, R211K

### Cloning of RT Sequence into a HXB2 Infectious Clone (RT_B′_ and RT_C′_ Virus)

Two *vectors* were used to clone different fragments of the wild type subtype B and C RT sequence into two molecular clones (pHXB2ΔRT and pHXB2ΔNRT) following the methodology previously described [14], [15]. The plasmid pHXB2ΔRT contains the complete genome of HXB2 except for a deletion between codons 25 and 554 in the RT gene. Two unique restriction sites, MluNI and NgoMIV, were inserted in this region through an adapter. The second plasmid, pHXB2ΔNRT, contains the HXB2 genome carrying a deletion of the N-terminal region of the RT connection domain (codon 25 to 315). It also contains two distinct restriction enzyme sites, MluNI and Van91I, which were inserted for cloning purposes.

The NL4-3 (subtype B) clone RT coding fragment was amplified with primers RT2569 and RT22 in the first PCR round (1744 bp), followed by RTball and NgoMIV-INT1rev in the second round (1623 bp) [13]. Therefore, the PCR product will contain the same flanking restriction enzyme sites, MluNI and NgoMIV, as in the vector pHXB2ΔNRT. To clone the full length RT, both the plasmid pHXB2ΔRT (codon 25 to 555) and the PCR product were quantified and digested with 10U of restriction enzyme MluNI at 37°C for 1 h. After digestion, products were re-purified and digested with 10 U of restriction enzyme NgoMIV for another 1 h at 37°C. Finally, the vector and the PCR products were ligated with the enzyme T4 DNA ligase (Invitrogen, USA) and digested with the restriction enzyme AspI to avoid re-ligation of original vector. For subtype C RT cloning, a sample from a naïve patient from the south of Brazil (C23) was amplified with primers RT2569 and 3′RTAA339 in the first PCR round (967 bp) and RTball and RT21 in the second round (941 bp) as previously described [14]. To clone the PCR fragment, we used the same strategy explained above, changing NgoMIV for Van91I in the PCR fragment digestion step. The ligation reaction was used to transform *E. coli* JM109 and HXB2 RTpNL 4.3 (RT_B′_) and HXB2 NRTC23 (RT_C′_), positive clones were purified by maxiprep (QIAGEN) for subsequent transfection into MT-4 cells to generate viral stocks. Several differences could be observed between RT_B′_ and RT_C′_ (V35T, E36A, T39D, K43R, S48T, Q102K, D121Y, K122E, C162S, K173N, Q174K, D177E, T200A, Q207E, R211K); however, none of these differences were previously associated with NRTI resistance (Table 1). All viral stocks were titrated through tissue culture infectious dose 50% (TCID_50_) prior to infection.

### *In vitro* Selection of Subtype B and C RT Infectious Clones (RT_B′_ and RT_C′_)

The initial drug concentration for the *in vitro* selection process was determined based on EC_50_ calculated for each inhibitor in our assays, which were 0.06 µM and 0.40 µM for ZDV and 3TC, respectively. Each chimeric strain was then used to infect 10^6^ MT-4 cells in sextuplicate using a MOI 0.002 spinoculation [16] generating 24 independent infections. Viral replication and efficiency of infection were monitored by light microscopy to observe cytopathologic effect (CPE) formation. In all samples, regardless of the viral clone in analysis, the cells were centrifuged after 5–6 days in culture, and 1 mL of the supernatant was used to re-infect fresh MT4 cells. The cells and three aliquots of infection supernatant were frozen at −70°C for further analysis. Drug concentration was maintained or increased two-fold based on cytopathologic effect after each passage. *In vitro* selection was stopped when drug cytotoxicity signs were evident. All collected aliquots were sequenced and analyzed for drug resistance mutation accumulation.

### Sequence Analysis (RT_B_, RT_C_, RT_B′_ and RT_C′_)

To check for the presence of drug resistance mutations in each passage, the first 225 codons of RT were amplified using specific primers (RT9-GTACAGTATTAGTAGGACCTACACCTGTC and RT12-ATCAGGATGGAGTTCATAACCCATCCA). The amplicons were purified, sequenced in an automated ABI3100 sequencer (Applied Biosystems) and edited manually using Seqman software (DNASTAR®). The genotypic interpretation of antiretroviral drug resistant mutations in RT was carried out through electronic submission to the Stanford database (http://hivdb.stanford.edu). ARV mutations were scored in all culture passages obtained from RT viral training.

## Results

**Figure 1:**
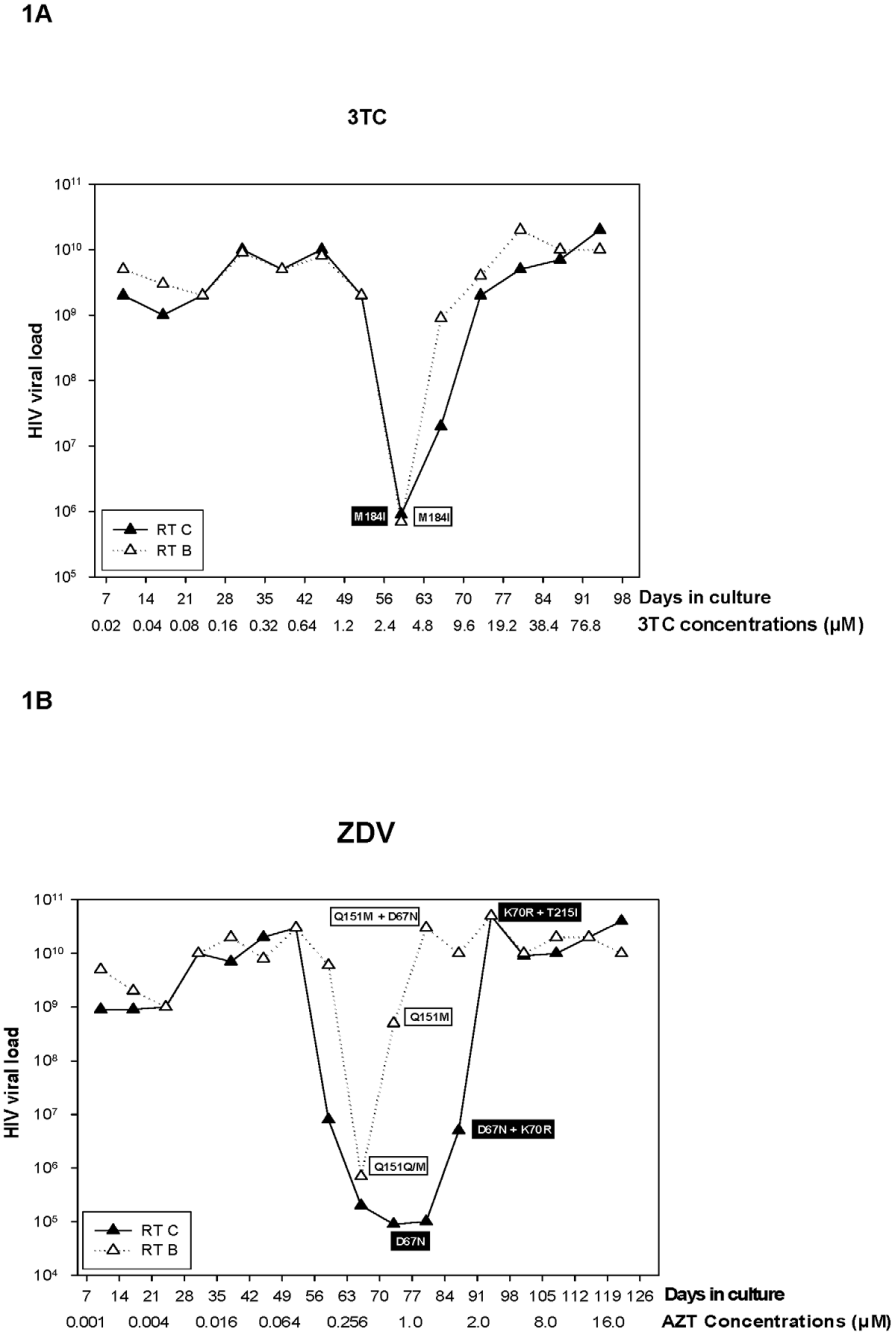
Graphics showing the selection process using 3TC (A) and ZDV (B) in MT4 cells infected with recombinant HIV-1 clones carrying the RT gene from subtype B and C. The virus load measured through real-time PCR from the culture supernatant is depicted on the y-axis. The time and concentration of the drugs utilized in each virus passage during the selection process are shown on the x-axis. Drug resistant mutations found in the clones during the passages are shown in the boxes below the curves. The white boxes contain mutations found in subtype B, and the black boxes contain the mutations found in subtype C.

To evaluate if the RT from different HIV-1 subtypes would acquire different resistance mutation patterns *in vitro*, we subjected recombinant viruses carrying either subtype B or C RT (RT_B_ and RT_C_ respectively) to increasing concentrations of ZDV or 3TC, and analyzed the acquisition of resistance mutations over time. In addition to that, HIV viral load was determined by real time PCR in culture, and correlated to the appearance of NRTI resistance mutations. Figures 1A and 1B depict the results of these analyses for 3TC and ZDV, respectively.

When 3TC was used to select for resistance in viruses carrying RT_B_ or RT_C_, the VL showed a clear change after 6 passages in increasing drug concentration (42 days, and 0.64 µM of 3TC). In fact, we could detect a 4-log decrease in the VL, which immediately rebounded to original levels and stayed constant all over the remaining passages. In the case of 3TC, both subtypes B and C behaved similarly and we could not observe any major difference in the kinetics. In contrast, the cultures under ZDV selection showed some differences in the VL kinetic between RT_C_ and RT_B_. The VL rebounded 14 days later in RT_C_ infected cultures when compared with the RT_B_ infected ones.

Several aliquots of culture supernatant were collected at the beginning and during the selection experiment (before VL rebound, in the middle of rebound time, and after VL recovery), viral RNA was isolated and the HIV-1 RT palm-finger region was sequenced. The rebound in 3TC selection was related to the appearance of a unique mutation M184I after 56 days (2.4 µM) regardless the subtype analyzed.

Contrasting to that, subtype B and C isolates followed a different mutational pathway under ZDV selection. The mutation Q151M was detected in subtype B clones after 63 days (0.256 µM), right after rebounding. This mutation was retained after subsequent passages. Additionally, the mutation D67N was incorporated after 77 days (1 µM), when the VL rebounded to original levels before the drug selection. Interestingly, subtype C followed a different route: it accumulated D67N after 77 days (1 µM) before rebounding and K70R after 84 days (2 µM) during the rebound process. The first mutation (D67N) was replaced by T215I at day 91 (2 µM), after the virus reached a VL level comparable to that before selection.

In order to check the reproducibility of these primary data, we cloned NL4-3 subtype B (RT_B′_) and C (RT_C′_) RT sequences into HXB2 background infectious clones. The infectious clones generated were transfected in MT4 cells and the supernatant was harvested after detection of cytopathologic effect (sincytium formation). The recombinant viruses were titrated and used to infect MT4 cells. The established infected cell culture was then selected with increasing concentrations of ZDV and 3TC in six independent experiments for each of the B and C clones.

In accordance to our previous results, 3TC selection yielded similar mutational profiles between RT_C′_ and RT_B′_, but with a slightly difference in kinetics. Mutation M184I appeared between 50–56 days (∼5–10 µM) of the selective process and was substituted by M184V or M184M/I/V at later passages with higher concentration of 3TC. The kinetics of M184I acquisition was similar for both RT clones. However, M184V was selected slight faster in RT_C′_ than RT_B′_. The only exception in the mutational profiles was the selection of the non-polymorphic mutation E203K in one replicate of clone RT_C′_ (Table 2). This mutation fluctuates between 44–96 days and was fixed with 104 days (1310 µM).

**Table 2:** Drug resistance mutations found during the *in vitro* selection of RTB_′_ and C_′_ with escalating concentration of 3TC.

3TC	RT_B′_ (1)	RT_B′_ (2)	RT_B′_ (3)	RT_B′_ (4)	RT_B′_ (5)	RT_B′_ (6)	RT_C′_ (1)	RT_C′_ (2)	RT_C′_ (3)	RT_C′_ (4)	RT_C′_ (5)	RT_C′_ (6)
Time (days)	Mutation (µM)	Mutation (µM)	Mutation (µM)	Mutation (µM)	Mutation (µM)	Mutation (µM)	Mutation (µM)	Mutation (µM)	Mutation (µM)	Mutation (µM)	Mutation (µM)	Mutation (µM)
6	NS	NS	NS	NS	NS	NS	NS	NS	NS	NS	NS	NS
10	WT (0.04)	NS	NS	WT (0.08)	NS	NS	WT (0.08)	(0.08)	(0.08)	WT (0.08)	NS	NS
16	NS	NS	NS	NS	NS	NS	M184M/I (0.16)	M184M/I(0.16)	M184M/I (0.16)	NS	NS	NS
20	WT (0.16)	WT (0.16)	WT (0.16)	WT (0.32)	WT (0.32)	WT (0.32)	M184M/I (0.32)	M184M/I (0.32)	M184M/I (0.32)	WT (0.32)	WT (0.32)	WT (0.32)
25	WT (0.32)	M184M/I (0.32)	M184M/I (0.32)	WT (0.64)	M184M/I (0.64)	M184M/I (0.64)		M184M/I (0.64)	M184M/I (0.64)	M184M/I(0.64)	M184M/I(0.64)	M184M/I (0.64)
31	M184M/I (0.64)	M184M/I (0.64)	M184M/I (0.64)	M184M/I (1.28)	M184M/I (1.28)	M184M/I (1.28)	M184M/I (1.28)	M184M/I (1.28)	M184M/I (1.28)	M184M/I (1.28)	M184M/I (1.28)	M184M/I (1.28)
38	NS	NS	NS	NS	NS	NS	NS	NS	NS	NS	NS	NS
44	M184M/I (2.56)	M184M/I (2.56)	M184M/I (2.56)	M184M/I (5.12)	M184M/I (5.12)	M184M/I (5.12)	M184M/I (5.12)	M184M/I (5.12)	M184M/I (5.12)	M184M/I; E203E/K* (5.12)	M184M/I (5.12)	M184M/I (5.12)
50	M184M/I (5.12)	M184M/I (5.12)	M184I (5.12)	M184I (10.24)	M184I (10.24)	M184I (10.24)	M184I (10.24)	M184I (10.24)	M184I (10.24)	M184M/I (10.24)	M184M/I (10.24)	M184M/I (10.24)
56	M184I (10.24)	M184I (10.24)	M184I (10.24)	M184I (20.48)	M184I (20.48)	M184I (20.48)	M184I (20.48)	M184I (20.48)	M184I (20.48)	M184I; E203E/K* (20.48)	M184I (20.48)	M184M/I (20.48)
62	NS	NS	NS	NS	NS	M184I (40.96)	NS	NS	M184I (40.96)	NS	NS	M184M/I/V (40.96)
69	M184I (40.96)	NS	NS	M184I (81.92)	NS	M184I (81.92)	M184I (81.92)	NS	M184I (81.92)	M184I; E203E* (81.92)	NS	M184M/I/V (81.92)
77	NS	NS	NS	M184M/I/V (163.84)	NS	M184M/I/V (163.84)	NS	NS	M184I (163.84)	NS	NS	M184M/I/V (163.84)
84	M184I (163.84)	M184I (163.84)	M184I (163.84)	M184M/I/V (327.68)	M184I (327.68)	M184M/I/V (327.68)	M184I (327.68)	M184I (327.68)	M184V (327.68)	M184I; E203E/K* (327.68)	M184I (327.68)	M184V (327.68)
90	M184I (327.68)	M184I (327.68)	M184I (327.68)	M184M/I/V (655.36)	M184M/I/V (655.36)	M184M/I/V (655.36)	(655.36)	(655.36)	NS	M184I; E203E/K* (655.36)	M184M/I/V (655.36)	NS
96	M184M/I/V (655.36)	M184I (655.36)	M184I (655.36)	M184M/I/V (1310.72)	M184M/I/V (1310.72)	M184M/I/V (1310.72)	M184M/I/V (1310.72)	(1310.72)	NS	M184M/I/V; E203E/K* (1310.72)	M184M/I/V (1310.72)	NS
104	M184M/I/V (1310.72)	M184M/I/V (1310.72)	M184M/I/V (1310.72)	M184V (2621.44)	M184V (2621.44)	M184V(2621.44)	M184V (2621.44)	M184V (2621.44)	M184V(2621.44)	M184M/I/V; E203K* (2621.44)	M184V (2621.44)	M184V (2621.44)
110	M184M/I/V (2621.44)	M184M/I/V (2621.44)	M184M/I/V (2621.44)	M184V (5242.88)	M184V (5242.88)	M184V (5242.88)	M184V (5242.88)	M184V (5242.88)	M184V (5242.88)	M184V; E203K* (5242.88)	M184V (5242.88)	M184V (5242.88)

NS - not sequenced,

* - mutation not associated with 3TC resistance, () - replicate number.

Confirming our previous results, ZDV selection generated different mutational patterns between subtypes B and C. Although the selection onset among replicates has temporal differences, the mutational profile is the same in the end. Contrasting to previous results observed in RT_B_, we could not find the multi-drug resistance mutation Q151M in the early stages of the selection process (figure 1B and Table 2).

The mutation D67N emerged before T215Y and F214L in some RT_B′_ replicates; however, in other RT_B′_ replicates D67N was selected after T215Y and F214L. Nevertheless, the final resistance profile was the same in all replicates: D67D/N, T215Y, F214L (Table 2). Three final resistance profiles were detected in RT_C′_ replicates: D67N, K70R and T215I (66,6%) or T215F (16,6%) or T215I/F (16,6%).The first mutations selected differ between replicates in RT_B′_ passages (Table 3). While RT_C′_ replicates accumulated TAM 2 pathway (K70R and T215F or T215I), RT_B′_ replicates followed TAM 1 mutations profile represented by T215Y. The mutation T215I (ATY or ATC) is an intermediary mutation between T215 (ACC) and T215F (TTT).

**Table 3:** Drug resistance mutations found during the *in vitro* selection of RT_B′_ and RT_C′_ with escalating concentration of ZDV.

ZDV	RT_B’_ (1)	RT_B’_ (2)	RT_B’_ (3)	RT_B_ (4)	RT_B’_ (5)	RT_B’_ (6)	RT_C’_ (1)	RT_C’_ (2)	RT_C’_ (3)	RT_C’_ (4)	RT_C’_ (5)	RT_C’_ (6)
Time (days)	Mutation (µM)	Mutation (µM)	Mutation (µM)	Mutation (µM)	Mutation (µM)	Mutation (µM)	Mutation (µM)	Mutation (µM)	Mutation (µM)	Mutation (µM)	Mutation (µM)	Mutation (µM)
1–44	WT (0.192)	WT (0.192)	WT (0.192)	WT (0.384)	WT (0.384)	WT (0.384)	WT (0.384)	WT (0.384)	WT (0.384)	WT (0.384)	WT (0.384)	WT (0.384)
50	NS	NS	NS	NS	NS	NS	WT (0.768)	T215T/I/F/S (0.768)	WT (0.768)	NS	NS	NS
56	WT (0.768)	WT (0.768)	WT (0.768)	WT (0.768)	WT (0.768)	WT (0.768)	D67D/N(1.536)	T215F(1.536)	D67D/N (1.536)	WT (1.536)	WT (1.536)	WT (1.536)
62	NS	NS	NS	NS	NS	WT (1.536)	D67D/N(1.536)	T215F(1.536)	D67D/N.K70K/R(1.536)	D67D/N (1.536)	NS	WT (1.536)
69	WT (3.072)	WT (3.072)	WT (3.072)	WT(3.072)	WT (3.072)	T215N/S/T/; F214F/L* (3.072)	D67D/N(3.072)	D67N; K70K/R (3.072)	T215F (3.072)	D67D/N; K70K/R (3.072)	WT (3.072)	D67D/N; K70K/R(3.072)
77	NS	NS	NS	D67D/N(6.144)	D67D/N (6.144)	D67D/N (6.144)	D67N; K70K/R; T215T/I(6.144)	D67N; K70K/R; T215T/I(6.144)	D67N; K70K/R; T215T/I(6.144)	D67D/N; K70K/R; T215T/I(6.144)	NS	D67D/N; K70K/R; T215T/I(6.144)
84	WT (6.144)	WT (6.144)	WT (6.144)	D67D/N(6.144)	T215Y; F214L* (6.144)	T215N/S/T/; F214F/L* (6.144)	D67D/N; K70R; T215T/I(6.144)	T215F(6.144)	D67D/N;K70K/R; T215T/S/I/F (6.144)	D67N; K70K/R; T215T/I(6.144)	WT (6.144)	D67N; K70R(6.144)
90	D67D/N;T215Y; F214L*(12.288)	D67D/N; T215T/N/S/Y; F214F/L*(12.288)	D67D/N; T215T/N/S/Y; F214F/L*(12.288)	D67D/N;T215Y; F214L*(12.288)	D67D/N; T215T/N/S/Y; F214F/L*(12.288)	D67D/N; T215Y;F214L* (12.288)	D67N; K70R; T215I(12.288)	D67D/N; K70K/R; T215F(12.288)	D67D/N; K70K/R; T215I/F (12.288)	D67D/N; K70K/R; T215T/S/I/F(12.288)	D67N; K70R; T215I(12.288)	D67N; K70R; T215T/I(12.288)
96	D67D/N;T215Y; F214L*(24.576)	D67D/N; T215Y; F214L*(24.576)	D67D/N; T215Y; F214L*(24.576)	D67D/N;T215Y; F214L*(24.576)	D67D/N; T215Y; F214L*(24.576)	D67D/N; T215Y;F214L* (24.576)	D67N; K70R; T215I(24.576)	D67D/N; K70K/R; T215F(24.576)	D67D/N;K70K/R; T215T/S/I/F (24.576)	D67N; K70R; T215T/I(24.576)	D67N; K70R; T215I(24.576)	D67N; K70R; T215T/I(24.576)
104	D67D/N;T215Y; F214L*(49.152)	D67D/N; T215Y; F214L*(49.152)	D67D/N; T215Y; F214L*(49.152)	D67D/N;T215Y; F214L*(49.152)	D67D/N; T215Y; F214L*(49.152)	D67D/N; T215Y;F214L* (49.152)	D67N; K70R; T215I(49.152)	D67D/N; K70K/R; T215F(49.152)	D67D/N;K70K/R; T215I/F(49.152)	D67N; K70R; T215I(49.152)	D67N; K70R; T215I(49.152)	D67N; K70R; T215I(49.152)
110	NS	NS	NS	D67D/N;T215Y; F214L*(98.304)	D67D/N; T215Y; F214L*(98.304)	D67D/N; T215Y;F214L* (98.304)	NS	NS	NS	D67N; K70R; T215I(98.304)	D67N; K70R; T215I(98.304)	D67N; K70R; T215I(98.304)

NS - not sequenced, * - mutation not associated with ZDV resistance, () - replicate number.

## Discussion

The selection of resistance mutations during antiretroviral therapy is associated with a reduction in drug susceptibility and viral fitness. Resistance-related mutations have been conventionally classified as primary or secondary based on their effect on drug susceptibility. While primary mutations reduce drug susceptibility and impact on replicative capacity, secondary mutations do not confer resistance by themselves but can enhance the replicative capacity and resistance levels of viruses carrying primary mutations.

The RT_B_ and RT_C_ recombinant clones showed the same VL levels (replicative capacity) in culture supernatant at the onset of training experiments (data not shown). However, when ZDV was added to the culture, a notable difference was observed in the replicative capacity. In this case, the kinetics of rebound and mutational patterns were distinct among RT_B_ and RT_C_ (figure 1B). Nevertheless, both clones behaved similarly when the same selection (M184I) was obtained using 3TC (figure 1A).

Our study has shown that both RT_B’_ and RT_C’_ treated with 3TC selected M184I in 8 weeks, but when selection continues until higher 3TC concentrations, we observed a shift from M184I to M184V (primary mutations). However, in some replicates, this shifting was incomplete and a mixture M184M/I/V was present at the end of the selection process. In addition, RT_C_’ appears to select M184V faster than RT_B’_ (Table 2), which is in accordance with previous reports [17]. This switch from isoleucine to valine could be due to the fact that M184I has a minor impact in RT processivity despite conferring 3TC resistance. Nevertheless, M184V has a major impact in both 3TC resistance and RT processivity. The selection of this mutation only under high drug concentrations tends to be advantageous for the virus. Moreover M184V brings an advantage for management of the treatment because the M184V-containing enzyme is less processive, decreasing the error of RT and consequently the frequency of mutations throughout the viral genome [18].

One of the RT_C’_ replicates treated with 3TC selected the E203K mutation (Table 2). This change, alongside with other mutations (K43E/N/Q, H208Y, and D218E), have already been associated with NRTI resistance; however, its actual impact in NRTI resistance has not been yet characterized [19], [20], [21].

Regarding the pattern of mutations selected with ZDV, we observed a difference in the profiles between RT_B_ and RT_C_. Clone RT_B_ started accumulating the mutation Q151M right after rebounding and this change was retained all over the culture. Furthermore, an additional mutation, D67N, was incorporated when the VL rebounded to original levels comparable to those before the onset of drug selection (figure 1B). Q151M is a primary mutation that *in vivo* can be co-selected together with a group of secondary mutations (A62V, V75I, F77L and F116Y) that confers a cross-resistance with all NRTIs. This complex, located around the catalytic site of the RT, is commonly referred to as “Q151M-mediated multinucleoside resistance” (Q151M-MNR) [22], [23]. Interestingly, no secondary mutation of Q151M-MNR was significantly evident upon selection with ZDV when the mutation Q151M was selected (RT_B_).

The additional selection experiments done in sextuplicate with RT_B’_ selected the T215Y TAM-1 pathway in all cases associated with F214L and D67N (Table 3). Although some researchers characterize D67N as a TAM-2 pathway mutation, this change has also been found in a TAM-1 background, in agreement with our data [24]. In addition, given that D67N does not impact on replicative capacity, this mutation could be selected first and then be replaced by T215Y, which has a major impact on ZDV resistance. Moreover, D67N could help to enhance RT processivity when selected after T215Y [25]. Stürmer M (2004), Ceccherini-Silberstein (2007) and F Puertas MC (2009) observed a negative association of F214L and T215Y, which was related to a decrease in replicative capacity and resistance if compared with viruses carrying only T215Y. However, these two mutations accumulate together in all RT_B’_ six replicates in our *in vitro* selection with ZDV [26], [27], [28].

Interestingly, the recombinant virus carrying RT_C_ followed a different route and initially accumulated D67N before rebounding and added K70R during the rebound process. The mutation D67N was replaced by T215I after the virus reached a VL value comparable to the levels before the onset of selection (figure 1B).

The additional six independent experiments with the RTc’ clone treated with ZDV selected the same TAM-2 pathway mutations (Table 2B). Nevertheless, the way that these mutations emerged was different between replicates. Some replicates initiated selection with T215F and others with the D67N and K70R mutations. However, three different final profiles with D67N and K70R mutations were observed in all replicates: T215I or T215F or T215F/I.

Essex M. et al. (2009) studied the impact of zidovudine resistance and thymidine analog mutations (TAMs) on subtype C HIV-1 replicative capacity and showed that the 67N and 70R accessory mutations gave an advantage over the WT in subtype C, but not in subtype B. They also showed that the TAM-2 mutant D67N/K70R/T215F had the slowest replication levels between both subtypes [25]. This might explain why D67N and K70R emerged first and then T215I was selected instead of 215F in 66% of replicates. Probably T215I has less impact on replicative capacity than T215F.

It is not known which factors cause the segregation of TAMs into two pathways. Whereas T215Y is one of the TAM-1 mutations firstly selected, that is not the case for T215F in the TAM-2 pathway. This can be probably explained considering the impact of these mutations on viral fitness [29]. Similarly our RT_B_’ data show that T215Y was the first mutation detected in TAM-1 pathway. However, RT_C_ and RT_C’_ data suggest that D67N and K70R were selected instead of T215F and then directed to TAM-2.

Our findings show that ZDV selected mutations in different RT subtypes belong to different TAM pathways. Whereas RT_B’_ mutations are related with TAM-1, RT_C’_ is related with TAM-2, in accordance with previous studies [30], [31], [32], [33]. However, other studies did not find this association in subtype C [34]. Still, these later studies were conducted in patients receiving highly active antiretroviral therapy (HAART), in which more than one drug is used. It is well known that drug combinations could influence the pattern of resistance mutations. Follow-up studies with patients infected with different HIV-1 subtypes receiving the same treatment should be done for better understanding of this phenomenon.

Of note, TAM-1 mutations are associated with an increase in phenotypic resistance when compared to TAM-2 ones [36], [37]. Furthermore TAM-1 is associated with cross-resistance to didanosine and tenofovir whereas TAM-2 remains susceptible [38]. These data combined with ours suggest that HIV-1 subtype C-infected patients may have larger chances of therapeutic success in ZDV-containing HAART regimens compared to those harboring subtype B.

The selection of drug resistant mutants during antiretroviral therapy could be a consequence of a complex interaction between the effect of a mutation on drug susceptibility and the effect of the mutation on the viral replication potential fitness. In this scenario, the polymorphism carried by different subtypes could influence the mutation pattern selected. This work was not designed to identify polymorphic sites found in subtype C RT that could be responsible for this different behavior in culture under drug pressure. Further experiments need to be done to clarify this point. The data shown here reveal that different subtypes can react differently to antiretroviral drug selection *in vitro* and suggest different odds of developing ARV resistance among patients infected with different HIV-1 subtypes.
